# Incidence and treatment outcomes of Graves’ disease in Thailand: a single-center retrospective observational study

**DOI:** 10.1186/s13044-022-00142-4

**Published:** 2022-12-19

**Authors:** Wasit Kanokwongnuwat, Nawarat Penpong, Chaninporn Sangsri

**Affiliations:** 1 Division of Nuclear Medicine, Department of Radiology, Prapokklao Hospital, 38 Leab Neon Road, Muang, Chanthaburi, 22000 Thailand; 2 Division of Endocrinology, Department of Medicine, Prapokklao Hospital, Chanthaburi, Thailand; 3 Division of Head Neck and Breast Surgery, Department of Surgery, Prapokklao Hospital, Chanthaburi, Thailand

**Keywords:** Hyperthyroidism, Incidence, Remission, Antithyroid drugs, Radioactive iodine, Surgery

## Abstract

**Background:**

Treatment patterns and outcomes of Graves’ disease (GD) are variable around the world. However, studies on treatment outcomes of GD from the Asian populations are limited. We aimed to evaluate treatment outcomes of GD in Thailand.

**Methods:**

Patients with new diagnoses of GD in a single center between 2014–2018 were retrospectively reviewed. The diagnosis of GD was based on clinical features, which included diffuse goiter, Graves’ orbitopathy (GO), pretibial myxedema and acropachy.

**Results:**

The age-adjusted incidence of GD was 26.57 per 100,000 per year. The study included 355 patients aged 15 years or above with a follow-up period of at least 24 months. Antithyroid drug (ATD) was the most popular first-line treatment modality with 98.7% patients receiving the treatment, followed by radioactive iodine (RAI) treatment in 1.3% patients. The most effective treatment modality was surgery with a remission rate of 100%. ATD had a lowest remission rate of 23.8%. Multivariable Cox regression analysis showed GO (HR 1.76, 95% CI 1.08–2.88) and initial TSH < 0.01 uIU/ml (HR 1.61, 95% CI 1.14–2.28) were significant factors associated with an increased treatment failure rate.

**Conclusion:**

Treatment failure with ATD in patients with GD was frequent in this population. The diagnosis of GD based solely on clinical features may explain the high treatment failure rate in this study. More definitive treatment could be used to prevent relapse and complications of the disease.

## Background

Graves’ disease (GD) is the most common cause of hyperthyroidism [1]. This disease occurs in 3% of women and 0.5% of men [2, 3] with an incidence of 20–40 cases per 100,000 population per year [3, 4]. It is an autoimmune disease caused by thyroid-stimulating hormone receptor antibodies (TRAb) rendering the thyroid gland to produce excess thyroid hormone. Symptoms of hyperthyroidism vary from mild to severe forms including weight loss, palpitation, anxiety, and heart failure [5]. Treatments of GD comprise antithyroid drugs (ATD), radioactive iodine (RAI), and surgery. ATD is generally considered the first-line treatment with a duration of treatment for 12–18 months [6]. Advantages of ATD include its easy accessibility and efficacy in achieving euthyroidism, but is associated with the risk of hepatotoxicity and agranulocytosis. ATD has a relatively low remission rate of 50–55%. RAI and surgery are often considered second-line treatment options and have a remission rate of above 90%. ATD is the most popular treatment modality for GD in Europe, Asia, and recently in the US [7–9]. In the past, RAI was the popular treatment in the US with 69% of the clinicians preferring the treatment modality [10], which declined to 59.7% in the 2011 survey study [7]. The likely contributors for this observation include concerns about new development or worsening of Graves’ orbitopathy (GO) and uncertainty surrounding the risk of radiation-induced malignancy following RAI. The 2016 American Thyroid Association (ATA) and 2018 European Thyroid Association (ETA) guidelines recommend RAI for patients who had side-effects from or relapse after ATD as well as those with severe co-morbidities [6, 11], but the recent National Institute for Health Care and Excellence (NICE) guideline in England recommends RAI as the first-line treatment due to its efficacy unless ATD is likely to achieve long-term remission [12]. The benefit and risk of each modality may affect each individual’s life differently, therefore the shared decision between doctors and patients is important [13, 14]. Few studies have analysed treatment patterns and outcomes in patients with GD in Thailand. A questionnaire survey suggested a trend toward the preference of ATD for treatment of GD [15] and a retrospective study from a private hospital showed a low remission rate following treatment with ATD [16]. This study aimed to explore the treatment choices and outcomes of GD in a suburban tertiary hospital.

## Methods

A retrospective study was conducted to include all newly diagnosed patients with GD treated in Prapokklao hospital, a tertiary hospital center in the Chanthaburi province, in the eastern part of Thailand, between 2014–2018. Patients were identified by searching electronic medical records (EMR) with at least 1 diagnosis code for GD (ICD-10-CM E05.0). The diagnosis of GD was based on clinical features, which included diffuse goiter, orbitopathy, pretibial myxedema, and acropachy. For incidence analysis, the patients at the age of 15 or more regardless of the follow-up time were included. For treatment analysis, the patients must have at least a 24-month follow-up after treatment and above the age of 15 years.

Data on age, gender, TSH, FT3, FT4, TSH-receptor antibodies (TRAb), treatment modality, and outcome were collected. The reference ranges were: 0.27–4.2 uIU/ml for TSH, 2.0–4.4 pg/ml for FT3, 0.93–1.71 ng/ml for FT4 and 0–1.75 IU/L for TRAb.

We used calculated dose for RAI. The patients had to withhold ATD for at least 7 days. Radioactive iodine uptake (RAIU) was measured at 24 h after the administration of 0.37 MBq of I-131 orally. The calculated dose (mCi) = thyroid gland weight (g) × 50–200 µCi/g x [1/24-h uptake in % of administered activity]. The calculated treatment dose was scheduled in the next day after measuring RAIU.

Treatment failure was defined as persistent hyperthyroidism or inability to withdraw ATD at the last follow-up visit with a follow-up period after treatment of at least 12 months for ATD, 6 months for RAI and 3 months for surgery. Remission was defined as either euthyroidism or hypothyroidism without ATD for at least 3 months.

Data analysis was performed using IBM SPSS statistics version 21. Univariable Cox regression analysis was used to explore factors associated with treatment failure with ATD. Variables with a *p-*value < 0.2 in univariable analysis were included in the multivariable analysis. Statistical significance was considered when a *p-*value was < 0.05. This study was approved by the institutional review board (COA No.094–2020).

## Results

### Incidence analysis

To analyse the incidence of GD, we only had the data on patients from the Muang district because the Thai healthcare system mandates patients to attend their local hospitals. The tertiary hospital received both patients based in the Muang district and those referred from other districts. Six hundred and twenty-seven patients were found after searching for newly diagnosed GD and 169 patients were from the Muang district. From the National Statistical Office of Thailand, there were 129,113 people in the Muang district of Chanthaburi province in the 10-yearly census carried out in 2020. The crude incidence rate of GD between 2014–2018 was 26.18 per 100,000 per year. A crude female to male ratio of 1.6:1. The standard population has a female to male ratio of 1.07:1, giving the same sex adjusted incidence of 26.18 per 100,000 per year. The age-adjusted incidence was 26.57 per 100,000 per year.

## Treatment analysis

**Fig. 1:**
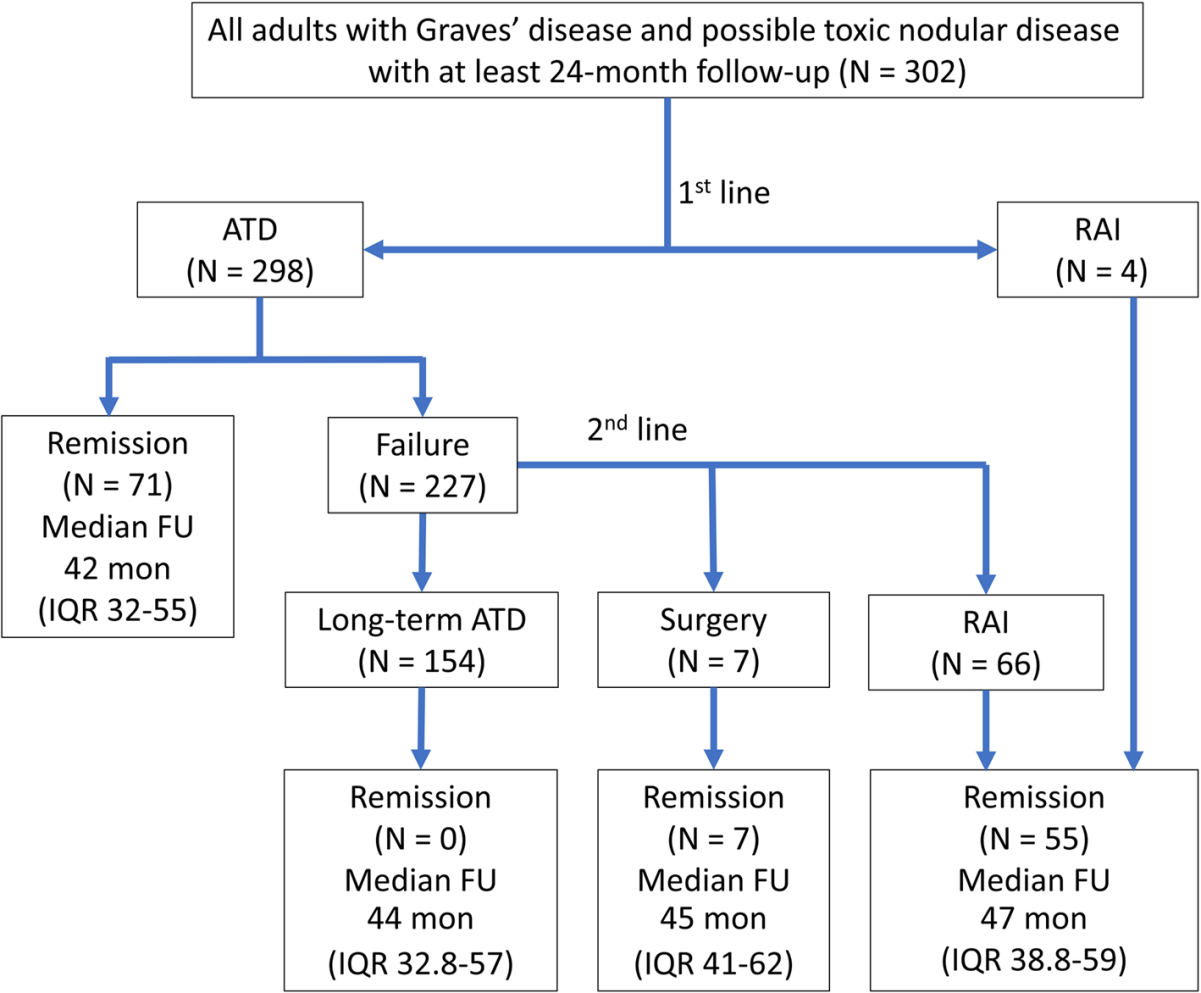
Study population treatment pathways. ATD, antithyroid drug; FU, follow-up; IQR, interquartile range

To analyse treatment outcomes, we excluded 325 cases (out of a total of 627) due to a follow-up period less than 24 months. The included cases in the treatment analysis were 302 patients (Fig. 1.). All patients had hyperthyroid symptoms with primary hyperthyroidism in thyroid function test. Extrathyroidal manifestations were presented with GO in 12.9%, thyroid acropachy in 0.3%, but no pretibial myxedema. The patients had thyrotoxic periodic paralysis in 4.6% and AF in 12.9%. Pregnancy occurred during the study period 2.6%. The patients had severe disease including impending thyroid storm and thyroid storm in 8.3%. 67.9% patients were female, a female to male ratio of 2.1:1. The mean age at diagnosis was 42.8 ± 15.1 years with a median follow-up period of 45 months (IQR 34–58) (Table 1.). Patients receiving ATD, RAI and surgery had median follow-up periods of 44 months (IQR 34–57), 47 months (IQR 38.8–59) and 45 months (IQR 41–62), respectively. All patients were diagnosed with GD based on clinical presentation and thyroid function tests. Only 2.3% patients had a TRAb test and 0.3% had a radioactive iodine uptake. There was a patient with nodular surface that proved to be Graves’ disease with a cold nodule by Tc-99 m pertechnetate thyroid scan.

**Table 1:** Clinical characteristics in overall treatments

Characteristics	Overall *N =* 302 (%)	Remission *N =* 133 (%)	Failure *N =* 169 (%)
Sex			
Female	205 (67.9%)	104 (78.2%)	101 (59.8%)
Male	97 (32.1%)	29 (21.8%)	68 (40.2%)
Mean Age (± SD) year	42.8 ± 15.1	42.6 ± 14.8	42.9 ± 15.5
Age group			
< 40 years	133 (44%)	61 (45.9%)	72 (42.6%)
≥ 40 years	169 (56%)	72 (54.1%)	97 (57.4%)
Thyroid function tests			
Median TSH uIU/ml	0.005	0.005	0.005
(N 0.27–4.2 uIU/ml)	(IQR 0.005–0.01)	(IQR 0.005–0.013)	(IQR 0.005–0.01)
Median FT3 ng/ml	14	13.8	14.8
(N 0.93–1.71 ng/ml)	(IQR 8–22.8)	(IQR 6.5–23.1)	(IQR 8.7–22.8)
Median FT4 pg/ml	4.6	4.3	5
(N 2.0–4.4 pg/ml)	(IQR 3.1–7.2)	(IQR 2.6–6.7)	(IQR 3.3–7.6)
TSH^a^			
< 0.01 uIU/ml	207 (68.5%)	86 (64.7%)	121 (71.6%)
≥ 0.01 uIU/ml	87 (28.8%)	42 (31.3%)	45 (26.6%)
FT3^a^			
≤ 3UNL	159 (52.6%)	74 (55.6%)	105 (49.3%)
> 3UNL	142 (47%)	59 (44.4%)	107 (50.2%)
FT4^a^			
≤ 3UNL	142 (47%)	68 (51.1%)	93 (43.7%)
> 3UNL	112 (37.1%)	44 (33.1%)	87 (40.8%)
Extrathyroidal signs			
GO	39 (12.9%)	17 (12.8%)	22 (13%)
Acropachy	1 (0.3%)	0 (0%)	1 (0.6%)
TPP	14 (4.6%)	5 (3.8%)	9 (5.3%)
AF	39 (12.9%)	16 (12%)	23 (13.6%)
Pregnancy	8 (2.6%)	2 (1.5%)	6 (3.6%)
Severe disease^b^	25 (8.3%)	10 (7.5%)	15 (8.9%)
Heart failure	17 (5.6%)	8 (6%)	9 (5.3%)
Hepatitis	2 (0.7%)	0 (0%)	2 (1.2%)

^a^Missing data in 8 pt of TSH, 1 pt of FT3 and 48 pt of FT4

^b^Including impending thyroid storm and thyroid storm

*UNL* Upper normal limit, *GO* Graves’ orbitopathy, *TPP* Thyrotoxic periodic paralysis, *AF* Atrial fibrillation

The chosen first-line treatments were ATD in 98.7% and RAI in 1.3%. ATD included methimazole (MMI) of 85.7%, propylthiouracil (PTU) of 1.3% and both MMI and PTU of 13%. ATD was used for a median duration of 36 months (IQR 26–50). All patients were treated with titration regime of ATD. Only 32.1% of the patients who failed with ATD as the first-line treatment proceeded to have surgery or RAI. The remaining patients continued long-term ATD. RAI was used in 23.2% of overall patients with a median cumulative dose of 333 MBq (9 millicurie) (IQR 222–555). There were 3.6% or 11 patients of overall patients who required multiple doses of RAI to achieve remission; 10 patients needed second doses and 1 patient needed a third dose. All patients who achieved remission from thyrotoxicosis with RAI became hypothyroidism. Surgery was carried out in 2.3% of overall patients including subtotal thyroidectomy in 4 patients and total thyroidectomy in 3 patients. There were 4 patient who chose RAI as the first-line treatment due to chronic hepatitis C infection in 1 patient, thyrotoxic periodic paralysis in 1 patient and agranulocytosis from ATD occurring less than 3 months after starting ATD in 2 patients. Patients who could not achieve remission after treatments, continued ATD to the end of the study. Overall remission at the last follow-up was 44%, and the highest remission rate was seen in patients treated with surgery (100%), followed by RAI (78.6%) and ATD (23.8%).

Serious adverse events of ATD included agranulocytosis (0.9% in the patients receiving ATD) without hepatitis or vasculitis. Amongst patients treated with surgery, 28.6% of all patients who underwent surgery had complications; 14.3% developed permanent hypocalcemia, 14.3% had transient hypocalcemia and none had recurrent laryngeal nerve injury. None of the patients who underwent RAI showed a complication of thyroid storm or worsening of Graves’ orbitopathy.

Multivariable cox regression analysis showed that a significant factor associated with time to failure in ATD patients was GO (HR 1.76, CI 1.08–2.88) and initial TSH < 0.01 uIU/ml (HR 1.61, CI 1.14–2.28) (Table 2.).

**Table 2:** Factors associated with time to failure in patients receiving ATD with univariable and multivariable cox regression analysis

Factors	Univariate *p-*value	HR (95% CI)	Multivariate *p-*value	HR (95% CI)
Sex				
Female	0.56	0.91 (0.67–1.25		
Male				
Age				
< 40y				
≥ 40y	0.39	1.15 (0.84–1.56		
GO				
Present	0.01	1.83 (1.13–2.95)	0.024*	1.76 (1.08–2.88)
Absent				
TSH				
< 0.01 uIU/ml	0.005	1.64 (1.16–2.33	0.007*	1.61 (1.14–2.28)
≥ 0.01 uIU/ml				
FT3				
≤ 3UNL				
> 3UNL	0.44	0.9 (0.72–1.33)		
FT4				
≤ 3UNL				
> 3UNL	0.3	0.84 (0.6–1.17)		
TPP				
Present	0.58	0.83 (0.42–1.62		
Absent				
AF				
Present	0.06	1.56 (0.99–2.45		
Absent				
Severe disease				
Present	0.52	0.84 (0.49–1.43		
Absent				

*GO* Graves Orbitopathy, *UNL* Upper normal limit, *TPP* Thyrotoxic periodic paralysis, *AF* Atrial fibrillation

## Discussion

We evaluated the incidence of GD in this local area of Thailand. The age-adjusted incidence of GD in this study was similar to other countries about 20–40 per 100,000, and the female to male ratio of 1.6:1 is lower than others showing up to 10:1 [1–4]. This study had high loss to follow-up which could be explained by referral issue. The patients were likely to go to their local hospitals after multiple visits at the referral hospital. The first-line treatment was an ATD, with 98.7% patients treated ATD, which is higher than a recent survey from Thailand showing 90.8% of the clinician responders favor the treatment [15] and a retrospective study from Thailand showing 70.8% of patients with GD receiving ATD [16]. The RAI treatment was used in 1.3% patients as a first-line treatment, which was less than in a previous study in which 21% of the GD patients were treated with RAI [16]. Prolonged ATD use was seen in this study, the duration was twice longer than the recommended treatment duration of 12–18 months [6]. Due to RAI unavailability in the local site, the leftover treatments were prolonged ATD treatment and surgery. This logistic problem probably explained the ATD preference and the duration of ATD treatment. MMI was the preferred ATD, owing to its availability and safety. PTU was usually preserved for use in severe disease and pregnancy. TRAb was checked in only 2.3% patients, due to high cost and long turn-around time.

The remission rate of ATD was 23.8%, similarly, the remission rate from the retrospective study of Thai patients in the private hospital was 30.7% [16]. This ATD remission rate was drastically different from the studies in the US and Europe of about 50% [17–19], even though ATD duration was prolonged. This could be partly explained by the fact that we used clinical diagnosis of GD in our study, and was not based on TRAb or thyroid uptake scan. Therefore, it is possible that some patients with a toxic nodule or a toxic multinodular goiter instead of GD were included in the study. We presume high iodine diet and genetic components may have contributed to this observation as well. Surgery showed the most effectiveness with remission rate. We used a median RAI dose of 333 MBq (IQR 222–555), this came from the calculated method. This dose was less than the dose recommended by the 2016 ATA guideline (370–555 MBq) and might explain the lower remission rate seen in our study [11]. The higher RAI activity could render higher remission rate when compared between 370 MBq vs 555 MBq [20].

Surgery showed the most remission rate but the high rate of complications. The complication might be a reflection of non-specialized surgeons in the countryside hospital where thyroidectomy is usually performed by general surgeons. The small sample size (7 out of 302 patients) of patients who underwent surgery could also cause the analysis of outcomes unreliable. Although, the referral to special teams dedicated for thyroidectomy with modern equipment should be considered, long-term ATD could be considered in a refractory disease [6]; this strategy showed better results compared to RAI in terms of hypothyroidism and GO [21, 22].

The remission rates in this study were 78.6% for RAI and 100% for surgery, less than those in the nationwide study in the US with 93% for RAI and 99% for surgery [17]. RAI had the best safety profile without any complications, except for the adverse effect of hypothyroidism. Surgery showed more serious complications of 14.3% with 1 out of 7 patients, followed by ATD of 0.9%. The risk of surgery was varied in previous studies, hypocalcemia occurred in 9.4–54.4% and recurrent laryngeal nerve injury occurred in 0.9–33% [23–26]. Risk of agranulocytosis occurred far less about 0.1–1.0% [27–29].

We analyzed the predictors of the failure rate and found GO and initial TSH < 0.01 uIU/ml as significant factors. We did not find any association between failure rate and FT3 and FT4. However, younger age, high FT3, FT4, orbitopathy, and large goiter size were significant factors predicting treatment failure in the past studies [16, 17, 30, 31].

Several limitations could have influenced this study. The incidence analysis included only a small number of patients due to poor data management. The retrospective study design might have contributed to confounders and bias. We used only clinical criteria for the diagnosis of GD and rarely performed TRAb test. This might have led to the inclusion of some patients with toxic nodular goiter and caused more treatment failure with ATD.

## Conclusion

Antithyroid drug was the preferred treatment modality for patients with Graves’ disease in Thailand; however, it is associated with less than 25% remission rate. The diagnosis of GD based solely on clinical features in this study may explain the high treatment failure rate. Thyroidectomy was effective but had relatively high prevalence of complication of hypoparathyroidism. RAI could be considered for definitive treatment which had moderate effectiveness with low complication.

## Data Availability

Datasets are available on request.

## Abbreviations

GD: Graves’ disease
ATD: Antithyroid drug
RAI: Radioactive iodine
TRAb: Thyroid-stimulating hormone receptor antibody
ATA: American Thyroid Association
ETA: European Thyroid Association
NICE: National Institute for Health Care and Excellence
EMR: Electronic medical records
TSH: Thyroid-stimulating hormone
FT3: Free triiodothyronine
FT4: Free thyroxine
MMI: Methimazole
PTU: Propylthiouracil
FU: Follow-up
IQR: Interquartile range
UNL: Upper normal limit
GO: Graves’ orbitopathy
TPP: Thyrotoxic periodic paralysis
AF: Atrial fibrillation
